# ChatGPT, Google, or PINK? Who Provides the Most Reliable Information on Side Effects of Systemic Therapy for Early Breast Cancer?

**DOI:** 10.3390/clinpract15010008

**Published:** 2024-12-31

**Authors:** Stefan Lukac, Sebastian Griewing, Elena Leinert, Davut Dayan, Benedikt Heitmeir, Markus Wallwiener, Wolfgang Janni, Visnja Fink, Florian Ebner

**Affiliations:** 1 Department of Gynaecology and Obstetrics, University Hospital Ulm, Prittwitzstr. 43, 89075 Ulm, Germany; 2 Department of Gynaecology and Obstetrics, University Hospital Marburg, Baldingerstraße, 35043 Marburg, Germany; 3 University Clinic and Polyclinic for Gynaecology, University Hosptial Halle (Saale), Ernst-Grube-Straße 40, 06120 Halle (Saale), Germany; 4 Department of Gynaecology and Obstetrics, Alb-Donau Klinikum Ehingen, Spitalstr. 29, 89584 Ehingen, Germany

**Keywords:** artificial intelligence, ChatGPT, Google, PINK, breast cancer, side effects, systemic therapy

## Abstract

**Introduction:** The survival in early breast cancer (BC) has been significantly improved thanks to numerous new drugs. Nevertheless, the information about the need for systemic therapy, especially chemotherapy, represents an additional stress factor for patients. A common coping strategy is searching for further information, traditionally via search engines or websites, but artificial intelligence (AI) is also increasingly being used. Who provides the most reliable information is now unclear. **Material and Methods**: AI in the form of ChatGPT 3.5 and 4.0, Google, and the website of PINK, a provider of a prescription-based mobile health app for patients with BC, were compared to determine the validity of the statements on the five most common side effects of nineteen approved drugs and one drug with pending approval (Ribociclib) for the systemic treatment of BC. For this purpose, the drugs were divided into three groups: chemotherapy, targeted therapy, and endocrine therapy. The reference for the comparison was the prescribing information of the respective drug. A congruence score was calculated for the information on side effects: correct information (2 points), generally appropriate information (1 point), and otherwise no point. The information sources were then compared using a Friedmann test and a Bonferroni-corrected post-hoc test. **Results:** In the overall comparison, ChatGPT 3.5 received the best score with a congruence of 67.5%, followed by ChatGPT 4.0 with 67.0%, PINK with 59.5%, and with Google 40.0% (*p* < 0.001). There were also significant differences when comparing the individual subcategories, with the best congruence achieved by PINK (73.3%, *p* = 0.059) in the chemotherapy category, ChatGPT 4.0 (77.5%; *p* < 0.001) in the targeted therapy category, and ChatGPT 3.5 (*p* = 0.002) in the endocrine therapy category. **Conclusions:** Artificial intelligence and professional online information websites provide the most reliable information on the possible side effects of the systemic treatment of early breast cancer, but congruence with prescribing information is limited. The medical consultation should still be considered the best source of information.

## 1. Introduction

Numerous new drugs have steadily improved survival in early breast cancer (BC) [1]. The most common form of systemic treatment for early BC is endocrine therapy [2]. Targeted therapies such as antibodies and immune checkpoint-, CDK4/6-, PARP-, or tyrosine kinase-inhibitors are also used. Nevertheless, 37% of patients still require chemotherapy (CTX) [3,4]. It is known that the burden of the diagnosis and prospect of CTX leads to fear, anxiety, panic, and other understandably negative reactions in many patients, as the side effects of systemic therapy cause changes in the quality of life [5]. In addition, patients with BC have been shown to seek further information around 1.5 times more frequently compared to other oncological patients [6]. This is known and evaluated as one of the most common coping mechanisms [7]. Despite educational counseling, the retention of information by patients remains very low due to the stress reaction [8,9]. Incorrectly understood information or misunderstanding can even lead to the rejection of CTX and, in extreme cases, compromise life expectancy [9,10]. It is therefore important that the information provided is correct, relevant, and congruent.

In addition to medical professionals, online media are a frequently used source of information [11]. Google is also one of the most frequently used search engines for medical questions [12]. Moreover, patients suffering from BC in Germany have the option of being prescribed an approved digital health application (DiGA) that provides supportive information and can thus improve their quality of life [13,14]. The providers of this DiGA offer additional information on their website about the side effects of systemic therapy for affected patients. Another form of information search is nowadays artificial intelligence (AI) in the form of chatbots. These explain the technical terms and formulate the information provided understandably and thus provide practical support for patients [15,16].

However, the question arises of how reliable these media are when it comes to providing information for patients about the side effects of systemic therapy for early BC.

## 2. Material and Methods

Three categories for digital media were identified in advance. The classic online internet search, the use of the app/website of the official provider of the DiGA, and the search via AI. Each digital medium was analyzed for the 5 most common side effects of the systemic therapy of early BC of each drug according to the best practice. The side effects were analyzed between August and September 2024.

### 2.1. Internet Research—Google

Google Search (Alphabet Inc., Mountain View, CA, USA) is an internet-based information retrieval service that uses algorithms to provide relevant websites, images, videos, and other content based on user input. The search on Google’s German website https://www.google.de (accessed on 15 August 2024) www.google.de was carried out in German. The query was carried out from an Apple computer with Ventura 13.4 as the system software in a single window of the web browser (Safari 16.5, Apple Inc., Cupertino, CA, USA), using a private mode setting, without logging in to Google services, and without allowing cookies. This was to exclude influences on the search function from previous searches, keywords, and cookies. The search region was set for Germany. In analogy to the classic Google search and based on the previous similar publications [17,18,19,20] and the recommendations of Google [21], the following input was used: “X most common side effects”, where “X” was the individual drug. The first 5 results given were then used for the analysis.

### 2.2. Website of DiGA Provider PINK!

In Germany, there are currently two DiGAs with legal approval for patients with BC and a focus on therapy: PINK! Coach (PINK gegen Brustkrebs GmbH, Hamburg, Germany) and Untire^®^ (Tired of Cancer B.V., Utrecht, The Netherlands). Untire focuses on fatigue and does not report side effects, and therefore was excluded. Other apps for patients with BC available in Germany (Meine Busenfreundin, Cankado etc.) are not approved as DiGAs and were therefore not included.

PINK! Coach is a therapy-accompanying DiGA for patients with BC from the time of diagnosis to the final follow-up. It aims to “strengthen health-related quality of life and health literacy as well as alleviate the psychological, psychosomatic and somatic consequences of breast cancer” [22]. The DiGA providers offer patients information on systemic treatment as additional information on their website https://pink-brustkrebs.de (accessed on 14 August 2024). Information on the side effects of the individual drugs can also be found here. This is offered as text, videos, and podcasts. The content of the website is also accessible to patients in the app and, according to PINK, it is identical to the website. Due to the simulation of the patient’s situation before the treatment decision, the evaluation was carried out according to the website information.

### 2.3. ChatGPT

The free and paid versions of ChatGPT were selected as the AI application. The AI ChatGPT (Open AI, San Francisco, CA, USA), which is publicly available in Germany, is currently one of the most widely used chatbots. In contrast to classic web searches, the input is made as a ’normal’ question. Using natural language processing based on the transformer architecture, the AI generates everyday language texts through probabilistic predictions based on patterns from the databases used in training [23]. For this reason, the freely accessible version 3.5 and the purchasable version 4.0 of the chatbot ChatGPT were used for the evaluation. The chat was opened anonymously and anew for each input, and no communication with ChatGPT took place after the input. Due to the chatbot function, the search query was analog to other studies [24,25,26] and formulated as follows: “What are the 5 most common side effects of X”. X was the particular drug. The responses of both versions were as follows: “The 5 most common side effects of X are:” and the chatbots named exactly 5 side effects for each drug.

#### 2.3.1. GPT 3.5

The free version ChatGPT 3.5 is a language model based on the GPT-3 model developed by OpenAI, which is based on a deep neural network with 175 billion parameters. It was trained on a large amount of text data using supervised learning to develop human-like text generation capabilities. The model uses transformer-based architecture to effectively process contexts and relationships in language and provide complex, coherent responses to queries.

#### 2.3.2. GPT 4.0

GPT 4.0 takes more parameters into account compared to 3.5, resulting in improved context processing and accuracy. In addition, GPT 4.0 shows a better performance with multilingual texts and more complex tasks, and offers enhanced machine learning capabilities [23].

### 2.4. Medication

Based on the recommendations of the Working Group of Gynecological Oncology [27] and the German breast cancer guidelines [2], a list of all the drugs approved for the systemic treatment of early BC (Table 1) was established. Ribociclib, which is expected to be approved for adjuvant use at the time of publication, was also included for completeness.

They were categorized into cytostatic drugs, endocrine therapeutics, and targeted therapies. As the reference source, the respective prescribing information, especially the ’most common side effects’ section of each drug, was used. For the study question, the first five side effects mentioned were reviewed. In the case of drugs with several manufacturers, all of the available prescription information was checked. This revealed no discrepancy in the side effects stated for a particular drug in different manufacturers.

### 2.5. Evaluation

Each digital medium reported up to five side effects for each of the twenty evaluated drugs. The ranking for each drug was performed as follows: 2 points if the side effect was mentioned correctly, 1 point if only the category of the side effect was mentioned without a specific side effect (e.g., gastro-intestinal side effects instead of nausea), and 0 points if the side effect was not reported. The maximum number of points was therefore mathematically 200 (5 side effects × 2 points × 20 medications). With this absolute maximum number of points per medium, a congruence score between the prescribing information and the respective digital medium was calculated in absolute terms (Y points) and as a percentage (congruence score = Y/200 multiplied by 100%). The digital media were then statistically compared in the overall result and in the subcategories of the medications. regarding their congruence score and achieved points. For example, for paclitaxel, the Google search reported the following side effects: nausea, stomatitis, diarrhea, and constipation. These were compared with the side effects summarized according to the prescribing information in Table 1. Therefore, for paclitaxel, Google received 2 points for nausea and 2 points for stomatitis, but no points for other side effects.

### 2.6. Statistical Analysis

**Figure 1:**
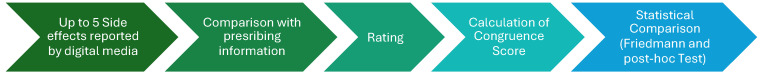
Summary of evaluation process.

The digital media were compared with the Friedmann test for non-parametric variables to evaluate the difference among four media. Thereafter, the post-hoc test with Bonferroni’s correction for multiple testing was performed to compare each pair, in order to detect significant differences between two particular media. All stated *p*-values are two-sided at a significance level of α = 0.05. The data were analyzed using IBM SPSS (Statistical Package for the Social Sciences) version 29 (SPSS Inc, Chicago, IL, USA). The evaluation process is summarized in Figure 1.

## 3. Results

**Figure 2:**
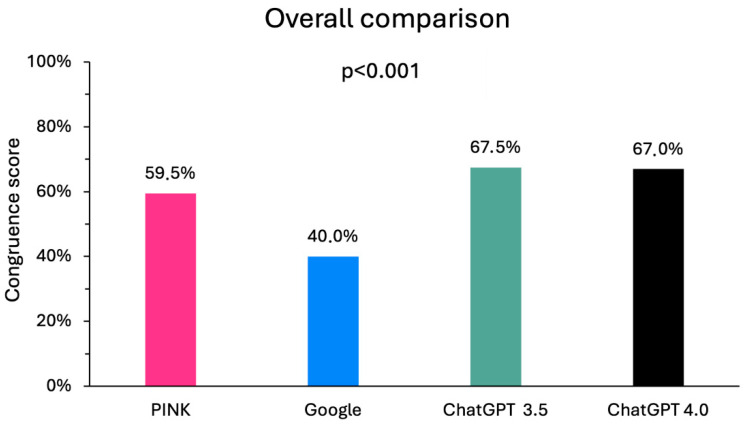
Overall comparison of the congruence of the digital media with the information for healthcare professionals on the side effects.

In general, only a moderate congruence with the prescribing information was achieved. The best score in the overall comparison was achieved by ChatGPT 3.5 with 67.5% (135 points), followed by ChatGPT 4.0 with 67% (134 points). The answers between the two software versions therefore differed in terms of content. Google scored 80 points (40.0%) in the overall comparison and PINK 119 points (59.5%). The differences in the total score between the digital media were statistically significant (*p* < 0.001; Figure 2), with the effect being mainly driven by the differences between ChatGPT 3.5 and Google (*p* = 0.005) and ChatGPT 4.0 and Google (*p* = 0.007).

**Figure 3:**
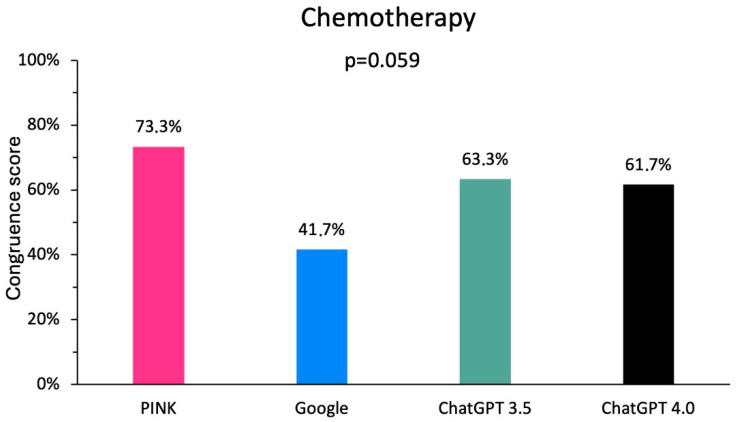
Comparison of the congruence of the digital media with the information for healthcare professionals on the side effects of chemotherapy drugs.

The subgroup analyses were carried out identically. Regarding chemotherapy, there was no significant difference among the evaluated digital media (*p* = 0.059), but a trend towards the dominance of PINK (Figure 3). ChatGPT 3.5 achieved 38 points (63.3%), ChatGPT 4.0 37 points (61.7%), Google 25 points (41.7%), and PINK 44 points (73.3%). However, the differences between the particular media were not significant according to the Bonferroni-corrected post-hoc tests.

**Figure 4:**
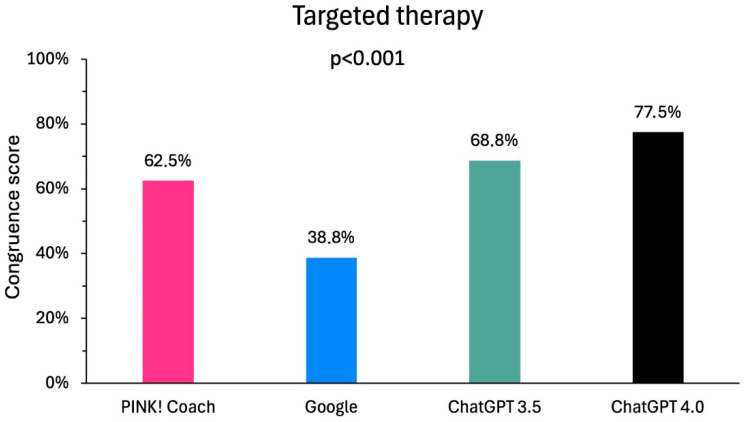
Comparison of the congruence of the digital media with the information for healthcare professionals on the side effects of targeted therapies.

There were significant differences in the targeted therapies (*p* < 0.001), which are shown in Figure 4. The best congruence score for targeted therapy was achieved by ChatGPT 4.0 with 62 points (77.5%), followed by ChatGPT 3.5 (55 points; 68.8%), PINK (50 points; 63.5%), and Google (48 points; 68.6%). When comparing the particular media, the difference between Google vs. Chat GPT 4.0 was statistically significant (*p* = 0.026).

**Figure 5:**
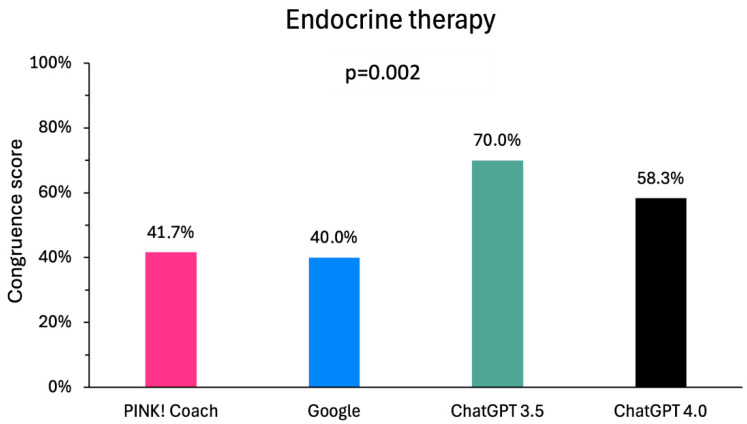
Comparison of the congruence of the digital media with the information for healthcare professionals on the side effects of endocrine therapies.

For the endocrine therapies, the ChatGPTs achieved the best congruence scores: ChatGPT 3.5 with 42 points (70%) and ChatGPT 4.0 with 35 points (58.3%). Google had 24 points (40.0%), and PINK had 25 points (41.7%). Overall, the congruence scores differ statistically significantly among each other (*p* = 0.002—Figure 5), but there are no significant differences between the particular media.

## 4. Discussion

To our knowledge, this study is the first to compare the reliability of information about chemotherapy side effects on Google, ChatGPT, and a DiGA information website. Digital media can offer only partially reliable information regarding side effects of the systemic treatment of eBC, as the results of our study show. Moreover, there were significant differences among the particular medias, with the dominance of ChatGPT.

Until 2022, The Health On the Net Foundation (HON) reviewed websites with medical content and issued a corresponding seal to ensure the quality and safety of the information [28]. This quality control has unfortunately been discontinued, and therefore it is necessary to prove the reliability of the digital media. A study by the Pew Research Centre in the USA shows that around 72% of the population searches for medical information via search engines such as Google [29], and only consult official medical sources as a second step. In the case of malignant diseases, the greatest need for information lies particularly in the side effects of treatment and prognosis [30]. Our results show that the congruence of the information provided by the digital media is only moderate, and in the best case, is just above two-thirds. The most recent development in the field of AIs, ChatGPT, showed the best agreement with the prescription information. A similar study as ours, but on renal cell carcinoma, showed comparable congruence scores for ChatGPT regarding the provided information [31]. Other studies confirmed that ChatGPT provides the correct basic information on the requested drug and also correctly clarifies common cancer myths [32,33]. However, these results confirm previous experience that, in everyday clinical practice, ChatGPT can be more supportive for the patient than for doctors [24,34]. This statement is supported by the results of other studies where it was shown that ChatGPT can advise patients more comprehensibly and even more empathetically than doctors on various issues [15,16,35,36].

Our data show that the correctness of the answers differs between the versions of ChatGPT, especially for information on endocrine and targeted therapies. The ChatGPTs achieved the best congruence scores in the general comparison, of 67% and 67.5%. The differences between the particular versions were not significant in the post-hoc test. However, it is known from previous studies that the individual versions have their strength in different medical areas [37], and even considering the same medical area, but in different topics, the responses can vary between ChatGPT 3.5 and 4.0 [38,39].

There are no studies that compared DiGA with Google and only a few studies that compared ChatGPT and Google for cancer information for patients. When searching for general information, etiology, and the pathogenesis of a disease, the advantage of Google was evident. However, questions about treatment options were not answered reliably by Google in previous studies, which is similar to our results [18]. Our results are in agreement with other studies that have also shown that ChatGPT is better than Google at providing medical information [40,41].

As far as the comparison with DiGAs, apps, or official websites is concerned, the study situation is limited. The first interventions to improve patient support were brochures [42] and, later, videos [43], which have proved to be effective. Now, with the era of digital media, the internet, and apps, new ways of providing information have become established. It has been shown that online-based supportive interventions can improve patients’ quality of life [44]. In our study, the PINK website achieved the highest congruence score for chemotherapy and numerically outperformed Google searches in each category. The data on the website are also available to patients in the official app, which can make access even easier. Thanks to the DiGa PINK! Coach and its website, patients with BC in Germany have complex support for their disease, which has a demonstrably positive effect on their well-being [13,14].

Supporting patients with information and symptom management should be an important part of the work with oncological patients [2]. Websites, apps, and artificial intelligence now offer ubiquitous and quickly accessible options for data transfer that can be used by patients at a low threshold [12,13,14,29,30]. This offers new options for supporting patients in the treatment of their disease. The first studies show that this support can even have a positive effect on survival [45].

The strengths of our study are that all currently commonly used drugs in the treatment of early breast cancer in Germany were systematically analyzed in all four modalities. We assessed the classical way by Google search, the modern way by artificial intelligence search, including both versions (affordable and freely accessible), and a unique method of web-based support from a DiGA provider. Our data provide information that is relevant for counseling sessions for both patients and doctors. Another advantage is the comparison with the prescribing information, where the most relevant side effects are listed. The limitation of this study is that the drugs have more than five side effects, which can also occur, so rarer side effects that were correctly mentioned by media could not be taken into account. Compared to Google and ChatGPT, the PINK! Coach’s website offers significantly more relevant information on treatment, that the methodology cannot reflect. Finally, there is also a lack of studies comparing medical consultations and digital media in terms of the quality of the information provided, meaning that the correlation of the data is only possible to a limited extent.

## 5. Conclusions

In summary, the digital media provide good, but only moderately congruent information with the specialized information on side effects of systemic therapy for early BC. There were differences in the respective subcategories, with the highest congruence score being achieved for targeted therapies. The artificial intelligence, ChatGPT, has achieved the best overall congruence score. However, the achieved score confirmed that the medical consultation should remain the most reliable source of information for the patient about side effects. However, this can be combined with other modalities to promote the retention of information.

## Figures and Tables

**Table 1:** Approved drugs for systemic treatment of early breast cancer with their five most common side effects according to prescription information.

	Drug	5 Most Common Side Effects				
Chemotherapy	**Epirubicin**	Myelosupression	Gastrointestinal disorders	Anorexia	Alopecia	Susceptibility to infection
	**Cyclophosphamide**	Myelosupression	Immunosuppression	Alopecia	Cystitis,	Microhaematuria
	**Docetaxel**	Myelosupression	Alopecia	Nausea, vomiting	Stomatitis, diarrhoea	Fatigue
	**Paclitaxel**	Myelosupression	Peirphere polyneuropathy	Arthralgia/myalgia	Allergy	Alopecia
	**Carboplatin,**	Myelosupression	Nausea/vomiting	Renal dysfunction	Liver value increase	Electrolyte disturbance (Na, K, Ca, Mg)
	**Capecitabine**	Gastrointestinal disorders	Hand-foot syndrome	Fatigue	Cardiotoxicity	Renal dysfunction
Targeted therapy	**Trastuzumab**	cardiac dysfunction	Infusion reactions	Haematotoxicity/neutropenia	Infections	Pulmonary side effects
	**Pertuzumab**	Diarrhoea	Alopecia	Nausea, vomiting	Fatigue	Neutropenia
	**Trastuzumab-emtasine**	Nausea	Fatigue	Musculoskeletal pain	Thrombocytopenia	Headache
	**Pembrolizumab**	immune-mediated reactions	Fatigue	Diarrhoea	Nausea	Infusion-related reactions
	**Abemaciclib**	Diarrhoea	Fatigue	Abdominal pain	Myelosupression	Nausea/vomiting
	**Neratinib**	Diarrhoea	Nausea/vomiting	Abdominal pain	Rash	Loss of appetite
	**Olaparib**	Nausea/ Vomiting	Fatigue	Anaemia	Diarrhoea	Loss of appetite
	**Ribociclib**	Neutropenia	Infections	Nausea/vomiting	Fatigue	Diarrhoea
Endocrine therapy	**Letrozole**	Hot flushes/ Sweating	Hypercholesterolaemia	Joint pain	Tiredness	Nausea
	**Anastrozole**	Headache	Hot flushes	Nausea	Skin rash	Joint pain
	**Exemestane**	Hot flushes	Joint pain	Tiredness	Nausea	Headache
	**Tamoxifen**	Nausea	Skin rash	Fluid retention	Hot flushes	Cycle changes
	**Leuprorelin**	Weight gain	Bone pain	Headache	Hot flushes/sweating	Loss of libido
	**Goserelin**	Hot flushes	Sweating	Loss of libido	Redness at the puncture site	Pain at the injection site

## Data Availability

The data that support the findings of this study are available from the corresponding author upon reasonable request.
